# Tailored lateral internal sphincterotomy (T-LIS) for chronic anal fissure by LigaSure Small Jaws©: a comparison with other non-conservative treatments for anal fissures

**DOI:** 10.1007/s13304-024-01943-y

**Published:** 2024-09-10

**Authors:** Maurizio Gentile, Vincenzo Schiavone, Antonio Franzese, Sebastiano Di Lascio, Nunzio Velotti

**Affiliations:** Department of General Surgery, Endocrinology, Orthopedics and Rehabilitation, Federico II University of Naples, Naples, Italy

**Keywords:** Anal fissure, Pain, Sphincterotomy, Bleeding, Bipolar device

## Abstract

An anal fissure is a small tear in the thin tissue (mucosa) that lines the anus. Anal fissures typically cause pain and bleeding with bowel movements. The cause is not fully understood, but low intake of dietary fiber may be a risk factor. Chronic anal fissure was defined as a split or ulceration in the posterior or anterior anoderm for at least 6 weeks: have distinct anatomic features such as muscle fibers visible in the wound. Anal fissures can be attributed to constipation or repeated straining: a hard fecal bolus cut the mucosa of anal canal that is relatively thigh at sphincter level management and optimal treatment of the disease is controversial. Many studies recommend conservative and medical treatment modalities as the initial treatment options since they are non-invasive and do not have risks such as anal sphincter injury. Lateral internal sphincterotomy (LIS) is considered the gold standard for treatment of chronic anal fissure. Nonetheless, anal incontinence is one of the worrisome complications of LIS. Fissurectomy is another option among those techniques which address the issues with LIS. LigaSure© (Valleylab) is a bipolar electrosurgical device designed to deliver high current and very low voltage to tissue. It monitors tissue impedance between the jaws of the instrument and continuously adjusts the delivery of energy. The use of LigaSure Small Jaw was never reported for anal fissures in literature. We have applied the use of this device to a group of patients complaining for chronic anal fissure in order to verify if there is any advantage to perform it compared to traditional technique (blade, scissors, electrocautery).

## Introduction

An anal fissure is defined as a longitudinal cut or crack in the distal anoderm which can extend from the anal verge to the dentate line. Anal fissures are a common cause of anal pain during, and for 1–2 h after, defecation. It can be very troubling because, if acute the severity of discomfort can exceed that which could be expected from a such trivial lesion [1]. The cause is not fully understood, but low intake of dietary fiber may be a risk factor. Anal fissures can be attributed to constipation or repeated straining: a hard fecal bolus cut the mucosa of anal canal that is relatively high at sphincter level. Based upon the causative possible factors, anal fissures are termed as primary or secondary. Primary fissures have no clear underlying cause and are likely to be related to local trauma, such as hard stools, prolonged diarrhea, vaginal delivery, repetitive injury or anoreceptive intercourse. Secondary fissures are found in patients with a history of previous anal surgical procedures, inflammatory bowel disease (e.g., Crohn’s disease), granulomatous disorders (e.g., tuberculosis, sarcoidosis), infections (e.g., human immunodeficiency virus, syphilis, *Haemophilus ducreyi*, herpes, cytomegalovirus) chemotherapy or malignancy (leukemia) [2]. Lockhart-Mummery believed that the explanation can be found in the structure of external sphincter, which is not circular in the lower portion [3]. Another theory that has been suggested is related to the blood supply of the area, which is already tenuous, but is further compromised by compression and contusion [4]. Midline is the most common location for primary fissures, while, anterior primary fissures, though rare, are more common in females. The cause of primary fissure is idiopathic. Acute fissures have a sharply fresh mucosal edges, heal spontaneously within three weeks with conservative medical management comprising a high fiber diet, warm sitz bath, and topical analgesic with steroids. Secondary anal fissures will not heal in any form of treatment until the primary cause is addressed. Chronic anal fissure was defined as a split or ulceration in the posterior or anterior anoderm for at least 6 weeks: have distinct anatomic features such as muscle fibers visible in the wound. Associated piles or hypertrophic papillae were not considered as complicating factors. Complicated anal fissures were defined as fissures associated with other anal symptoms, such as fistulas, abscess or hemorrhoids. These fissures often need surgical treatment [5]. Basically, treatment for anal fissure usually comprises reducing the sphincter pressure with physical or chemical methods. Studies on the methods of treatment of chronic anal fissures range from medical applications to surgery. There is no general agreement on ideal therapy for chronic anal fissures. Management and optimal treatment of the disease are controversial. Many studies recommend conservative and medical treatment modalities as the initial treatment options since they are non-invasive and do not have risks such as anal sphincter injury [6, 7]. The American Society of Colon and Rectal Surgeons (ASCRS) recommends conservative treatment as the initial treatment choice which includes stool softeners, high fiber diet and warm sitz bath. However, a significant number of patients do not respond this conservative treatment. Further treatment options will therefore be required [7, 8].

Lateral internal sphincterotomy (LIS) is considered the gold standard for the treatment of chronic anal fissure. Nonetheless, anal incontinence is one of the worrisome complications of LIS. In most series, LIS seems to be the operation of choice, resulting in success rates as high as 96–100% with high patient satisfaction [9]. Fissurectomy is another option among those techniques which address the issues with LIS. Some studies showed that patients with chronic fissures who are refractory to medical treatment responded well to fissurectomy [10].

LigaSure (Valleylab) is a bipolar electrosurgical device designed to deliver high current and very low voltage to tissue. It monitors tissue impedance between the jaws of the instrument and continuously adjusts the delivery of energy. Hemorrhoidectomy by LigaSure electrosurgical unit seems to be very effective treatment and results in better surgical outcomes when compared with the conventional excisional hemorrhoidectomy. [11]. The use of energy devices, such as LigaSure^TM^ scalpel, has reshaped the concept of hemorrhoid surgery and in turn, improved patient outcomes and simplified the work of surgeon. The possibility to perform a virtually bloodless operation makes the operation easier, quicker and safer, thus justifying the increased cost of the LigaSure™ device compared to diathermy [12, 13].

The use of LigaSure Small Jaw was never reported for anal fissures in literature before. We have applied the use of this device to all tailored sphincterotomy in order to reduce postoperative bleeding [13].

## Methods

A group of 918 patients from January 2016 to June 2022 with non-conservative treatments for anal fissures were considered. All patients were treated in our institution as proctological day-surgery patients. All patients have a 6-month follow-up. All failed with previous conservative therapies with dietary modifications, proper hygiene, topical nitrates or calcium channel blockers for a minimum 6-week period. The mean duration of symptoms in the whole group was 16.2 (range 4–120) months.

Duration of symptoms for at least 6 weeks is the most important factor for the AF diagnosis type of AF (acute vs chronic), which is the most important factor which guides the therapeutic plan. A written informed consent form was obtained from the patients when they were first diagnosed with anal fissure. Patients were informed about the treatment: further treatment options and probable complications were explained. They were prepared with enema at home and a single dose of cefazolin were administered 30’ before skin incision. Patient demographics are in Table 1: 510 patients with chronic anal fissure underwent to tailored LIS ± excision of piles, 202 patients with complicated anal fissures underwent anal fissurectomy (AF), 183 Botulinum Toxin local infiltration (BT) and 23 anal dilatation (AD).

**Table 1:** Characteristics of study population

Overall	T-LIS	BT	AF	AD	*p* value
918	510	183	202	23	
Age^a^, (mean + SD) (years)	44.8^a^, 14.1	44.2^a^, 13.9	46.4^a^, 14.5	46 ± 11.3	0.42
Male	214 (43%)	102 (56%)	78 (38.6%)	19 (82.6%)	0.001
Female	296 (57%)	81 (44%)	124 (61.6%)	4 (17.3%)	0.001
Short-term incontinence	43(8.4%)	6 (3%)	27 (13.3%)	11 (47.8%)	0.001
Gas incontinence	10 (4%)	6 (3%)	27 (13,3%)	11 (47.8)	0.001
Liquid incontinence	5 (2%)	4 (2%)	1 (1%)	9 (39.13%)	0.001
Solid incontinence	2 (1%)	2 (1%)	0	2 (8.69%)	0.001
Long-term incontinence	27 (5.2%)	2 (1.09%)	5 (6%)	6 (26.08%)	0.001
Gas incontinence	27 (5.2%)	2 (1.09%)	5 (6%)	6 (26.08%)	0.001
Liquid incontinence	8 (1.5%)	0	3	2 (8.69%)	0.001
Solid incontinence	0	0	0	0	
Healing rate (9 weeks)	484(94.9%)	127(69.39%)	138(68.3%)	7 (30.4%)	
Recurrence rate	26 (5%)	56(30.6%)	64 (31.6%)	16 (69.5%)	0.001
Bleeding	34 (6.6%)	0	23 (11.38%)	1 (4.34%)	

^a^https://www.sciencedirect.com/science/article/pii/S101595842100244X?via%3Dihub#tbl1fna

### Tailored LIS with LigaSure SJ

Patients were in lithotomy position. A perianal anesthetic infiltration was performed using a solution of bupivacaine and lidocaine. The anal canal was inspected and Eisenhammer retractor was placed. The left intersphincteric groove was identified at 3 o’clock position. A small incision was made in the left intersphincteric groove above the intersphincteric groove with LigaSure just to allow to the forceps to enter in the space and rotate pointing the dentate line, isolated the sphincter between the branches of the device, pressed two or three times the muscle with LigaSure Small Jaws to seal, and finally sectioned the one-third to one-half of internal sphincter muscle to the level of the dentate line. Usually the wound is bloodless and is closed. The patients were discharged on the same day with follow-up in clinic after 2 weeks and then 3 months until the anal fissure was healed.

Assessment was obtained by the performing surgeon. Follow-up data were obtained for a time period of 6 months. Healing was based on symptomatic relief and fissure healing confirmation.

### Botulinum injection

The patients were injected with BTX type A (Botox, Allergen). A total dose of 10–20 U was diluted in 1 ml isotonic saline and injected while the patient was lying on his/her left side. An insulin syringe and a (26 G) needle were used for injection. The injection was done in the internal anal sphincter. Half of this dose was injected on both sides of the fissure.

### Fissurectomy

Anal dilatation was done for four minutes using a four-finger technique followed by fissurectomy. The fissure was excised using a scalpel, and the wound was curated till a healthy margin was reached up to the level of the internal sphincter. Thus, a fresh ulcer was made without scar tissue and was allowed to heal by secondary intention. The presence of any concomitant skin tag or sentinel pile was also excised.

### Anal dilatation

Anal dilatation was performed before surgery using a dilator with diameter of 33 mm. Dilator was lubricated with water-based sterile gel before inserted into anal canal. After anesthesia agent was administered and the patient was relaxed, the dilator was gently inserted into anal canal and rectum. The dilatation process was maintained for 20 min.

### Outcomes

The outcomes were reported according to Boland [14] and Jin [18]: (1) healing at 8–9 weeks (2) Recurrence overall, (3) temporary incontinence to gas, liquid or solid, (4) permanent incontinence to gas, liquid or solid, and (5) bleeding. The mean time was recorded just for the T-LIS with LigaSure in order to verify the speed of the procedure.

### Statistical analysis

Statistical analysis was performed using the SPSS 26 system (SPSS Inc., Chicago, IL, USA). Continuous data were expressed as the means ± standard deviation (SD), and categorical variables were expressed as the % changes. The chi-square χ^2^ test was used to analyze categorical data, and the Mann–Whitney test was used to analyze continuous variables. All results are presented as two- tailed values with statistical significance defined as *p* values < 0.05.

## Results

As reported in Table 1, there is a little difference between male and female gender, but it is not significant. Similarly, there is no difference in age in different groups of patients.

Of 510 patients who underwent to T-LIS, about 95% was healed within 9 weeks (484 pts) from treatment compared to 127 (69%) of the BT and 138 (68%) in AF group. The lowest rate of healing was in AD group with 7 out 23 patients (30%). A significant difference is in onset and percent of recurrence between the first three groups and anal dilatation in which about 70% of patients is early involved. In the LIS group, the overall recurrence over a period of 6 months for all patients was 26 patients (5%) compared with 56 (30,6%) of BT and 64 (31%) for AF and 16 (69%) in AD. Concerning the incidence of complications, 17 patients (6%) complained about transitory incontinence after T-LIS and 9 (1.7%) reported permanent flatus incontinence. A higher incidence of complication was observed in AD group with 47% of transitory incontinence and 26% of permanent flatus incontinence. Perianal bleeding was observed in 34 patient (6.6%) in T-LIS LigaSure group, 21 under anticoagulant therapy, vs 64 (31.6%) in AF. No adverse reaction were registered. The mean time for the LigaSure procedure was recorded: 4′24″ (range 4′–8′35″). Finally, the cost of a single device in Italy is about 400 € (280–450) and the compensation for the procedure in day surgery from the National Italian Health System is 100,700 €.

## Discussion

Anal fissures probably begin by the tearing of the mucosa during defecation. Hard stools are most commonly implicated, but explosive liquid stools can produce the same results. This starts a vicious cycle of pain, causing spasm in the anal sphincter, which results in increased friction during defecation and leads to further tearing and pain. Anal fissure has a negative effect on patients’ quality of life. In fact, pain can impair the social life and ability to work and often leads to refusal to defecate or postponement because of the fear of intense.

Currently, ischemia is considered the most likely cause for development of an anal fissure. There is a paucity of anal blood vessels, especially in the posterior midline, and it is believed that anal spasm further reduces blood flow [15]. Although many acute anal fissures with a fresh skin tear heal spontaneously, some do not. Those that do not develop secondary changes with raised edges exposing the white, horizontally oriented fibers of the internal sphincter (chronic fissure) and prolonged anal pain. There are many treatments for AF, some involving chemical sphincterotomy agents, such as nitrate donors, for example, glyceryl trinitrate (GTN) or isosorbide dinitrate. Other medications that are thought to work topically on the area include diltiazem and nifedipine. Other treatments include injecting botulinum toxin (Botox) into the internal anal sphincter. More invasive treatments include lateral sphincterotomy, a procedure where the lowest fibers of the internal anal sphincter are cut. This reduces anal tone in that area, allowing the AF to heal. Other treatments that are less well-studied include the use of advancement flaps, fissurectomy, and other variations of lateral sphincterotomy, including tailored sphincterotomy and anal dilatation [16–18].

The main goal of sphincterotomy is to increase the blood flow of the anoderm by decreasing the maximum anal sphincter pressure by 18–50%. This technique provides an improvement between 82% and 100% of cases [19, 20].

The ASCRS Clinical Practice Guidelines report that lateral internal sphincterotomy is associated with consistently superior healing rates compared with medical therapy for chronic anal fissure and even “may be offered in select patients without first confirming failure of pharmacological treatment.” (Grade of Recommendation: Strong recommendation based on high-quality evidence [21]).

Multiple randomized trials confirmed better results of lateral internal sphincterotomy (LIS) compared with medical treatments, such as topical nitrates, calcium channel blockers, or botulinum toxin, with 88–100% healing rates and an incidence of fecal incontinence from 8 to 30% [19, 20]. One reason for the superior results associated with LIS may be the poor compliance associated with long-term medical therapy, an observation that was confirmed by a recent Cochrane review comparing surgical and nonsurgical therapies for anal fissures [9]. Quality of life has also been reported as improved in patients undergoing surgery. Better results are reported in patients with no previous fecal incontinence of any grade: it could mean to avoid to perform a sphincterotomy in patients with IBD and women with obstetric injuries [21–23]. 6% of participants who were randomized to lateral sphincterotomy reported a degree of fecal or flatus incontinence. The reported incidence of fecal incontinence after lateral sphincterotomy varies in literature, between 6 and 33%, and is not consistent with the rates from this study.

Continence scores are significantly correlated with the extent of the division, and the proportion of patients with a continence score of 0 was significantly greater in patients in whom sphincter division was less than 25%, which for women in this study corresponded to less than 1 cm [24].

Lateral sphincterotomy was the most effective treatment for healing of AF, while botulinum toxin as some medical treatments were comparatively less effective as reported by Jing [18]. There is an insufficient number of studies to compare the relative effectiveness of other less well-studied treatments, such as anal fissurectomy and controlled anal dilatation.

LIS is also superior to manual anal dilatation that is uncontrolled [25]. It was compared in two controlled trials to fissurectomy, but results were better for sphincterotomy in terms of healing rate and nonsignificant for incontinence. The gradual improvement in pain in the AF group as compared to immediate pain relief in the LIS group should not be regarded as a main difference between the two procedures since all patients were eventually pain-free within 1 week of the operation. To emphasize the results, no patient in the LIS group suffered from incontinence to flatus. There was no fissure recurrence in this group during the follow-up period. In total, 96.6% reported satisfactory results with their operation [26]. In Davies [27], the success rate for fissure healing following surgery was 92%, being significantly more likely in patients with textbook symptoms (*p* = 0.016) and those with chronic disease (*p* = 0.006). In descending order of frequency, botulin toxin and anal fissurectomy reported a good rate of healing compared to anal dilatation and other medical treatments where the success rate was significantly lower [28]. The safe extent of division in women treated for chronic anal fissure with high anal resting pressure and no symptoms of fecal incontinence is less than 25% of the total sphincter length, which in women corresponds to less than 1 cm as reported by Regadas [29]. Finally, Mousavi, in 2009 reported the surgical treatment of chronic anal fissure not responding to conservative management. LIS may be the better treatment and, perhaps, the preferable surgical technique with fewer total complications (*p* < 0.005) [17].

Finally, the cost is significantly higher in our group, but the operation time and bleeding are reduced even not significant. Moreover, the cost of the device is covered by the state refund. The possibility to perform a virtually bloodless operation makes the operation easier, quicker and safer, thus justifying the increased cost of the LigaSure™ device compared to diathermy. LigaSure was significantly advantageous over conventional technique in reducing risk of complications and operation time as well as perioperative and postoperative blood loss. The reduction of operative times resulted in decreased operating room occupancy costs, but the overall cost of surgery was significantly higher in LigaSure group.

## Conclusions

Lateral internal sphincterotomy is the treatment of choice for anal fissure after failure of conservative treatment. Better results were reported comparing to other surgical techniques, such as fissurectomy and non-conservative treatments. LigaSure SJ is a bipolar device which allows performing the procedure in a reduced time and in a safer way even if differences with traditional techniques are not significant. The costs of the operation are higher in LigaSure group.

## Data Availability

The cost of the device is 300-400 Euros and Italian reimburse is about 1000 Euros for each procedure.
